# Longitudinal characterization of COVID-19 across a surveillance transition in Japan: thirteen epidemic waves, 2020–2025

**DOI:** 10.1038/s41598-026-51317-0

**Published:** 2026-05-21

**Authors:** Chihiro Wakabayashi, Masayoshi Matsumoto, Shiho Sato, Yui Nakaizumi, Yuri Matsubara, Daisuke Matsubara, Yoshihisa Aiba, Izumi Oki, Yosikazu Nakamura, Hiroshi Yanagawa

**Affiliations:** 1 Department of Health Sciences, Saitama Prefectural University, 820 sannomiya, Koshigaya, Saitama, Japan; 2 Department of Public Health, Dokkyo Medical University School of Medicine, Mibu, Tochigi, Japan; 3 Division of Community and Family Medicine, Center for Community Medicine, Jichi Medical University, Shimotsuke, Tochigi, Japan; 4 Utsunomiya Public Health Center, Utsunomiya, Tochigi, Japan; 5 Japan Kawasaki Disease Research Center, Tokyo, Japan

**Keywords:** Diseases, Health care, Mathematics and computing, Medical research

## Abstract

Long-term comparison of COVID-19 epidemic waves is challenging because surveillance systems and reporting practices change over time. In Japan, official reporting shifted repeatedly between January 2020 and December 2025, from comprehensive nationwide notification to restricted reporting and sentinel-based surveillance. Using publicly available national data on reported cases and deaths, we characterized 13 epidemic waves while explicitly accounting for reporting transitions. We described weekly incidence and mortality rates per 100,000 population using population-based indicators to enable comparison across heterogeneous surveillance frameworks. Early waves showed very high observed case fatality ratios. Observed case fatality ratios declined markedly in subsequent waves, although estimates varied with reporting criteria and adjustments for reporting limitations, including periods when mild cases were excluded. Death counts were available only through week 18 of 2023, precluding evaluation of population-based mortality and observed case fatality ratios after the transition to sentinel-based surveillance. This transparent bridging framework enables longitudinal, comparability-oriented description of 13 epidemic waves in Japan across the 2023 surveillance transition. These findings should be interpreted as descriptive patterns observed under evolving surveillance contexts, and causal attribution is beyond the scope of this study.

## Introduction

In Japan, the legal reclassification of COVID-19 to Category 5 in May 2023 fundamentally changed the surveillance architecture, shifting from comprehensive case notification to sentinel-based reporting. This transition disrupted the longitudinal interpretability of epidemic trends because post-transition data are not directly comparable to earlier nationwide notification data. Most existing analyses of COVID-19 trends in Japan have therefore focused on the pre-transition period. In this study, we propose a transparent bridging framework to enable continuous, comparability-oriented description of epidemic waves across the surveillance transition, allowing long-term characterization through 2025 using publicly available national data.

Since the confirmation of the first COVID-19 case in mid-January 2020 in Japan^1^, 13 epidemic waves were observed through December 2025. This study aims to describe trends and epidemiological patterns of COVID-19 epidemic waves in Japan, and to describe how these trends evolved alongside the implementation of various preventive measures, as well as to provide basic data for advancing effective programs against potential outbreaks of unknown infectious diseases in the future.

Following the confirmation of the first case of COVID-19, the Japanese government implemented various measures, including revision to the Act on the Prevention of Infectious Diseases and Medical Care for Patients with Infectious Diseases (the Infectious Diseases Control Law)^2^. In February 2020, COVID-19 was designated as the Designated Infectious Disease under the Infectious Disease Control Law and further designated as a quarantinable infectious disease under the Quarantine Law. Quarantine measures were initiated in response to the outbreak on the international cruise ship ‘Diamond Princess’^3,4^. Furthermore, in April 2020, the government issued a state of emergency declaration under the Act on Special Measures Against Novel Influenza^5^, enabling requests that residents refrain from going out and other intensive non-pharmaceutical measures.

Subsequently, effective 13 February 2021, COVID-19 was legally positioned as a “Novel Influenza and Other Emerging Infectious Disease” under the Infectious Diseases Control Law (i.e., treated as equivalent to Category II), providing a legal basis for measures such as recommendations for hospitalization. This legal classification targets infectious diseases considered highly transmissible and potentially severe. As a result, all medical institutions were required to report the number of new cases on a daily basis. Japan promoted risk reduction messaging centered on avoiding the “three Cs” (closed spaces, crowded places, and close-contact settings), alongside other non-pharmaceutical interventions. From 26 September 2022, after confirming nationwide establishment and strengthening of health follow-up centers, Japan narrowed the scope of mandatory physician notifications under the Infectious Diseases Control Law. Thereafter, notifications were required only for cases in four groups: adults aged ≥ 65 years, patients requiring hospitalization, high-risk patients for whom clinicians judged COVID-19 therapeutics or newly initiated oxygen therapy to be indicated, and pregnant persons^6^.

On 5 May 2023, the World Health Organization announced that COVID-19 no longer constituted a Public Health Emergency of International Concern^7^. In Japan, policy also shifted in light of reduced risks of severe disease and hospitalization. From week 19 of 2023, COVID-19 was reclassified as a Category 5 infectious disease, comparable to seasonal influenza, and legally enforceable requests and interventions were discontinued, with prevention relying primarily on individual decision-making and voluntary measures. Key changes included ending requests for self-isolation of cases and close contacts, expanding the range of medical institutions able to provide consultations, and returning health-insurance cost-sharing to standard levels^8^.

Because Japan modified its countermeasures and surveillance systems in response to the evolving pandemic situation, it became difficult to assess long-term trends in reported cases and deaths using consistent criteria. By adjusting reported data to account for changes in surveillance practices, this study aimed to improve comparability across epidemic waves and enable continuous observation of trends from January 2020 to December 2025. This study is expected to serve as fundamental information for preparedness against future pandemics.

## Methods

### Data sources and surveillance phases

We conducted a descriptive analysis of time-series changes in COVID-19 incidence and mortality in Japan using publicly available national surveillance data released by the Japanese government. The study period covered mid-January 2020, when the first COVID-19 case was confirmed in Japan, through December 2025. Data on reported new COVID-19 cases and deaths were obtained from official governmental sources^9^. Throughout the study period, case reporting criteria changed in accordance with policy decisions and healthcare capacity. Up to week 38 of 2022, all newly diagnosed COVID-19 cases were notified nationwide under the Infectious Diseases Control Law. From week 39 of 2022 to week 18 of 2023, national notification was restricted and did not include mild cases, with reporting limited to cases meeting predefined clinical or risk-based criteria. From week 19 of 2023, after COVID-19 was reclassified as a Category 5 infectious disease, routine nationwide notification was discontinued and surveillance shifted to a sentinel system, in which approximately 5,000 fixed medical institutions (primarily influenza sentinel sites) reported weekly COVID-19 case counts within the national Infectious Disease Surveillance Survey^10^.

### Bridging of the post-transition period

To support longitudinal, comparability-oriented comparison of weekly incidence rates (per 100,000 population) across surveillance phases, we derived bridged national case estimates for the sentinel-surveillance period using an overlap calibration approach. During the four-week overlap (weeks 15–18 of 2023), nationwide case counts and sentinel reports were released in parallel. We calculated a conversion factor by relating the nationally reported case totals to the contemporaneous sentinel-based counts expressed per sentinel site; based on this overlap, weekly national case counts after week 19 of 2023 were estimated by multiplying the reported cases per sentinel site by 55. These bridged national case estimates were then converted to weekly incidence rates per 100,000 population using official population statistics. This bridging step was designed to facilitate continuity of descriptive comparison under changing reporting systems and is not intended to provide precise estimates of the true number of SARS-CoV-2 infections.

### Outcome measures

Mortality rates were calculated as reported COVID-19 deaths per 100,000 population. Observed case fatality ratios (CFRs) were calculated as reported deaths divided by reported cases for each epidemic wave. For wave-based calculations of deaths and CFRs (Table 3), weekly death counts were available only through week 18 of 2023; therefore, wave-specific deaths and CFRs were calculated up to wave 9. Values marked ** in Table 3 indicate adjusted case estimates for periods with reporting limitations.

**Table 3:** Reported COVID-19 cases, deaths, and observed case fatality ratio by epidemic wave (waves 1–9).

Wave no.	Duration of the wave	No. of new cases	No. of deaths	Observed case fatality ratio (deaths/reported cases) (%)
1	2020, weeks 19–22 (2020 May 6 - June 2)	1,414	296	20.93
2	2020, weeks 23–38 (2020 Jun 3 - Sep 22)	58,946	673	1.14
3	2020, weeks 39–2021, weeks 7 (2020 Sep 23–2021 Feb 23)	318,264	7,045	2.21
4	2021, weeks 8–23 (2021 Feb 24 - Jun 15)	316,577	6,200	1.96
5	2021, weeks 24–46 (2021 Jun 16 - Nov 23)	871,825	3,485	0.40
6	2021, weeks 47–2022, weeks 23 (2021 Nov 24–2022 Jun 14)	6,363,465	12,979	0.20
7	2022, weeks 24–39 (2022 Jun 15 - Oct 4)	10,983,009	15,063	0.14
8	2022, weeks 40–2023, weeks 11 (2022 Oct 5–2023 Mar 21)	12,000,889	27,586	0.23
		19,201,422*		0.14*
9	2023, weeks 12–46 (2023 Mar 22 - Nov 19)	210,165	552	0.26
		336,264*		0.16*

Reported cases, deaths, and observed case fatality ratios are summarized for Waves 1–9, for which death counts were available in the case-based series.

Note: The observed case fatality ratio was calculated as (reported deaths)/(reported cases) for each epidemic wave. In Wave 8, reported case counts reflect the reporting criteria at that time, which excluded mild cases. Values marked with * indicate adjusted estimates of reported cases to account for reporting limitations; the corresponding adjusted case fatality ratios (also marked with *) were computed using the adjusted case counts and the same reported death counts. Death data were available only through week 18 of 2023; therefore, Wave 9 deaths and ratios were calculated using weeks 12–18 of 2023 only, although the wave duration shown reflects weeks 12–46.

### Epidemic wave definition

We defined epidemic waves using the national weekly incidence time series. For each wave, the start week was defined as the first week of a sustained increase following a local minimum; the peak week was defined as the week with the maximum incidence within that interval; and the end week was defined as the week after which incidence returned to a subsequent local minimum and did not show a sustained increase for at least 2 consecutive weeks. When plateaus or minor shoulders were present, we applied the same operational rule consistently across all waves and resolved ties by selecting the earliest week meeting the peak criterion.

## Results

### Epidemic waves and incidence trends

**Fig. 1:**
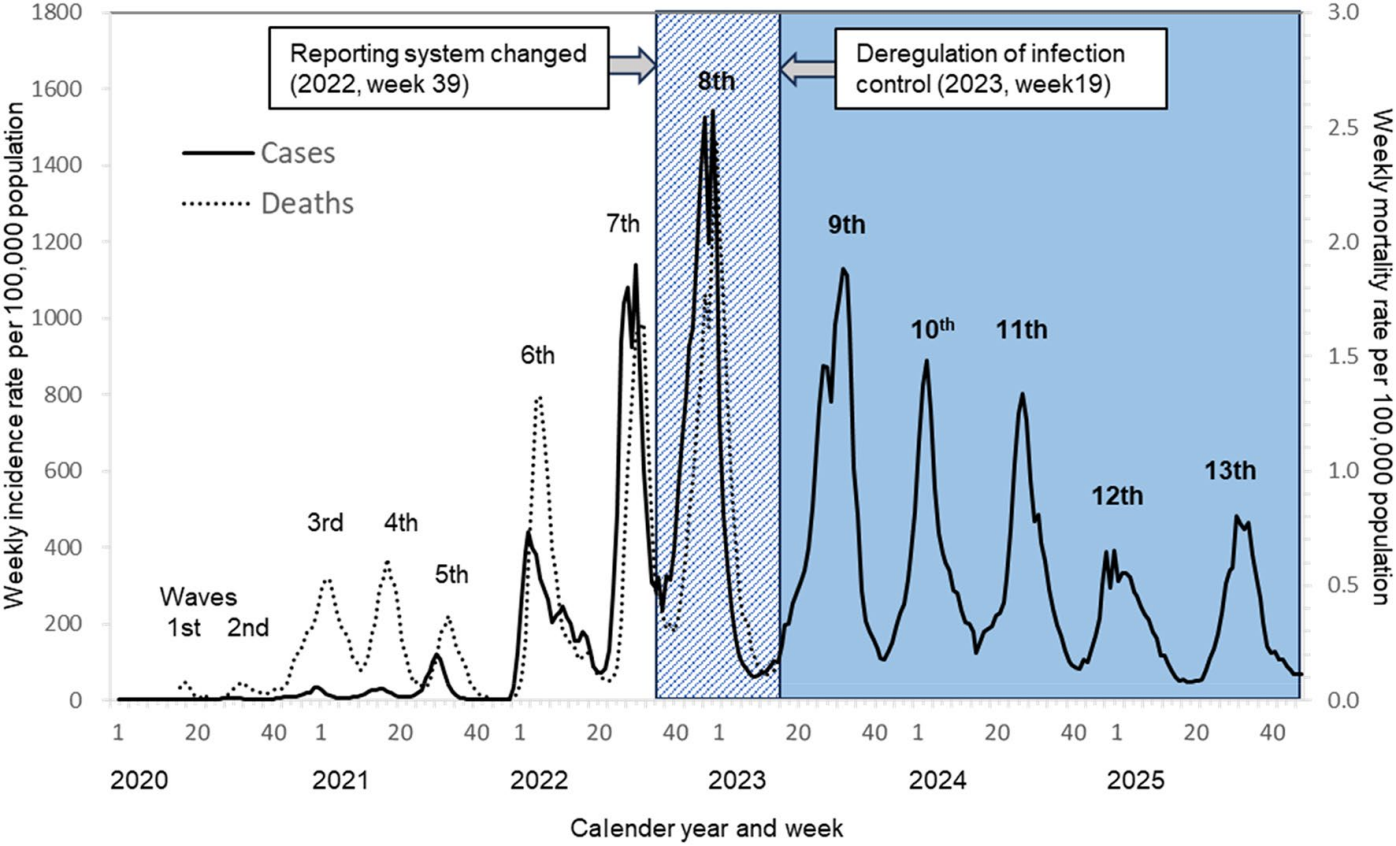
Weekly COVID-19 incidence and mortality rates in Japan, 2020–2025. Weekly incidence and mortality rates per 100,000 population are shown from January 2020 to December 2025, and 13 epidemic waves are indicated. The case-reporting system changed over time from nationwide notification to restricted reporting and subsequently to sentinel-based surveillance. Mortality data were available only through week 18 of 2023.

Figure 1 shows the weekly incidence rate of COVID-19 per 100,000 population in Japan from January 2020 to December 2025. During this period, 13 epidemic waves were identified. The first wave occurred in early 2020, followed by successive waves with increasing peak heights until the eighth wave. After the transition to sentinel-based surveillance in week 19 of 2023, five waves (Waves 9–13) were observed; however, Wave 9 overlapped the transition period, and peak incidence was generally lower than that observed during earlier waves. In the early phase of the pandemic, particularly during the first and second waves, increases in incidence were relatively modest in absolute numbers but were followed by sharp rises in subsequent waves. The largest incidence peaks were observed on the eighth wave, after which peak levels gradually declined. Despite changes in surveillance systems over time, incidence trends supported continuous observation of epidemic dynamics across the entire study period.

Through week 18 of 2023, the mortality-rate curve broadly mirrored the incidence-wave profile; however, the timing and magnitude of changes differed across waves. Mortality increased during Wave 3 and then declined slightly in Wave 5. Thereafter, mortality rose sharply from Wave 6 onward, with a steep upward trend continuing through Waves 7 and 8. Mortality data were available only through week 18 of 2023; therefore, mortality trends after that time are not shown.

**Fig. 2:**
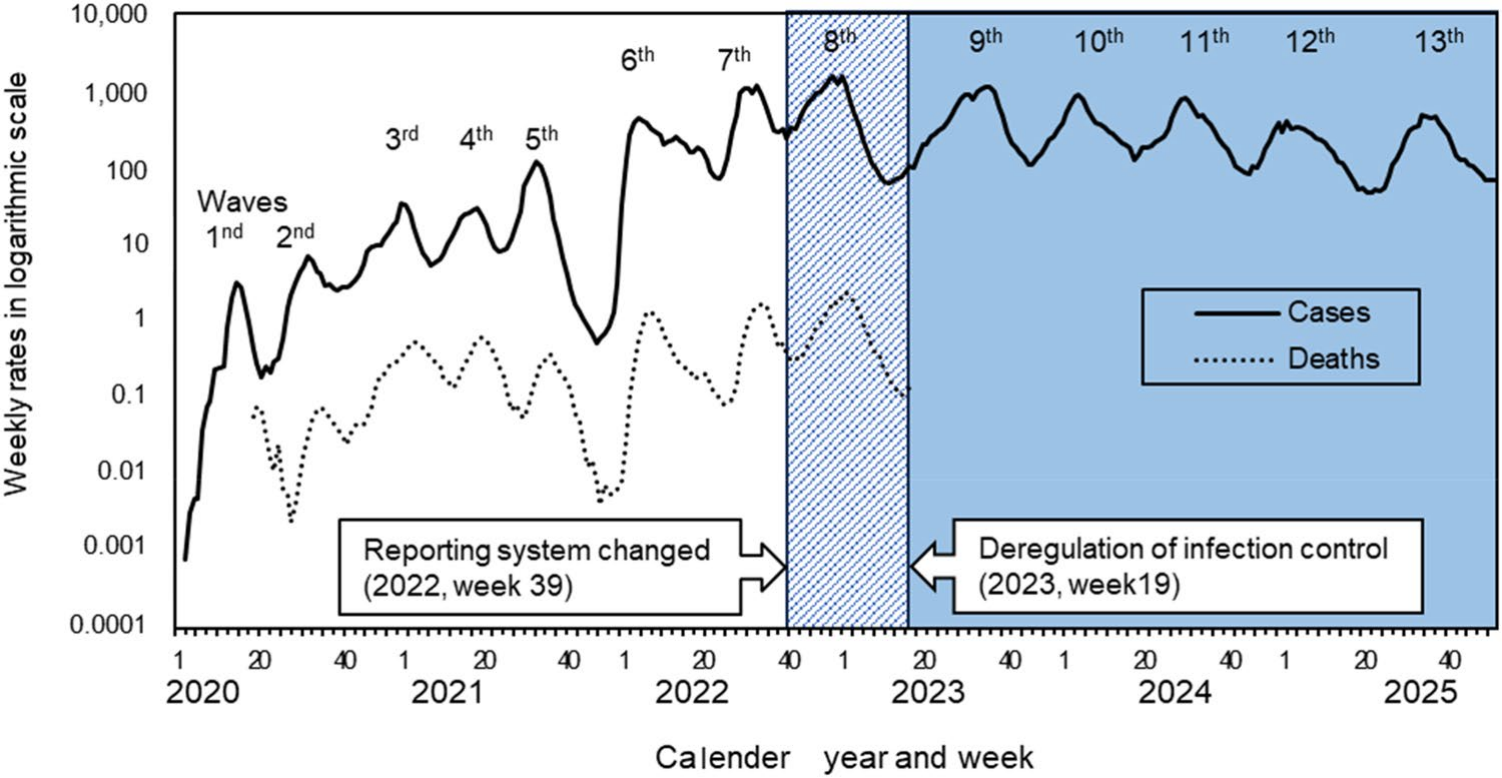
Epidemic wave profiles of COVID-19 incidence and mortality on a logarithmic scale. Weekly incidence and mortality rates per 100,000 population are shown on a logarithmic scale to facilitate comparison of epidemic wave magnitude and shape across 13 waves in Japan.

Figure 2 shows epidemic wave profiles of weekly COVID-19 incidence and mortality rates per 100,000 population plotted on a logarithmic scale. The logarithmic presentation of incidence and mortality highlights the differences in scale between early and later epidemic waves. This visualization underscores the importance of considering magnitude differences when interpreting long-term epidemic trends, rather than indicating changes in intrinsic disease severity.

### Variants, vaccination coverage, and epidemic waves

Table 1 summarizes the dominant SARS-CoV-2 variants, vaccination coverage, and major epidemiological characteristics across epidemic waves^11^. Early waves were dominated by the ancestral strain and Alpha variant, whereas later waves corresponded to the emergence and predominance of Delta and Omicron variants. Vaccination coverage increased substantially from the fourth wave onward, coinciding with changes in epidemic patterns, including lower mortality rates despite high incidence^12^.

**Table 1:** Major SARS-CoV-2 variants and vaccination status across 13 COVID-19 epidemic waves in Japan, 2020–2025.

Wave No	Epidemic waves and major mutant strain		Vaccination administered in each wave				
	Duration of the Wave	Main variants (%)	The *n*^th^ vaccination	Vaccination uptake	Time at vaccination	Persons eligible for vaccination	Vaccine strain
1	2020, weeks 19–22 (2020 May 6 – June 2)	B.1.1 (92.8), B.1.1.284(4.2), B.1(1.5)	-	-	-	-	-
2	2020, weeks 23–38 (2020 Jun 3 – Sep 22)	B.1.1.284 (75.2), B.1.1.214 (22.8), B.1.1(1.5)	-	-	-	-	-
3	2020, weeks 39–2021, w7 (2020 Sep 23–2021 Feb 23)	B.1.1.214 (75.3), B.1.1.284 (17.2), R.1(3.8), B1.1.7(1.3)	Original SARS-COV-2 strain started in week 7 (February 17), 2021 as special vaccination program in Vaccination Law by National Expense				
4	2021, weeks 8–23 (2021 Feb 24 – Jun 15)	B.1.1.7 (76.9), R.1 (16.2), B1.1.214(4.1)	1st	80.5%	2021, week7~2021, week9~	(1) Health care workers (2) Aged 65 + (3) General	Original SARS-COV-2 strain
5	2021, weeks 24–46 (2021 Jun 16 – Nov 23)	AY.29 (77.8), B.1.1.7 (15.5), AY.29.1(4.5)	2nd	79.5%			Original SARS-COV-2 strain
6	2021, weeks 47–2022, w23 (2021 Nov 24–2022 Jun 14)	BA.1.1.2 (41.4), BA1.1(8.7), BA.2.3.1(7.6), BA.2(5.8), BA.2.29(5.0), BA2.24(4.7), BA.2.3(4.6), BA2.3.13(3.8), BA2.10(3.1), AY29(2.8)	3rd	67.1%	2021, week 49~	Aged 12y+Aged 5y+ (after 2022 Feb)	Original SARS-COV-2 strain
7	2022, weeks 24–39 (2022 Jun 15 – Oct 4)	BA.5.2(24.2),BA.5.2.1(15.1), BF.5(14.0), BA.5.1(3.5), BA.2, BA.5.2.12(3.1)	4th	46.2%	2022, week16 ~	Aged 5y+	After week 36,2022: Bivalent Omicron BA.1.
8	2022, weeks 40–2023, w11 (2022 Oct 5–2023 Mar 21)	BF.5 (22.4), BA.5.2 (12.5), BA.5.2.1 (10.6), BQ1.1(5.5), BA5.2.6(3.9)	5th	30.4%	2022, week 40~	Aged 6 m+	After week 40,2022: Bivalent Omicron BA.4/5
9	2023, weeks 12–46 (2023 Mar 22 – Nov 19)	XBB.1.16 (7.5), EG.5.1 (6.9), EG.5.1.1 (6.7)	6th	20.3%	2023 week18~	Aged 6 m+	Bivalent Omicron BA.1 or BA.4/5. After week 40, 2023: monovalent Omicron XBB.1.5
10	2023, weeks 47–2024, w18 (2023 Nov 20–2024 May 5)	JN.1(11.9), XDQ.1 (7.2), KP.3 (7.0)	7th	14.0%	2023 week36~	Aged 6 m+	Monovalent Omicron XBB.1.5
11	2024, weeks 19–45 (2024 May 6 – Nov 10)	KP.3.3(41.2), KP.3.3.3 (16.9), KP.3.1.1 (4.3)	Special vaccination program in Vaccination Law by National Expense finished in week 13 (March 31), 2024				
12	2024, weeks 46–2025, w23 (2024 Nov 11–2025 Jun 8)	XEC (22.9), KP.3.1.1 (8.9), XEC.4(5.8), XEC.2(5.4), KP.3.3(5.1)	-	-	-	-	-
13	2025, weeks 24–2025, w50 (2025 Jun 9–2025 Dec 14)						

This table summarizes the timing and duration of 13 epidemic waves, together with predominant variants and the status of the national COVID-19 vaccination program during each wave.

### Surveillance transitions and longitudinal comparability

Throughout the study period, surveillance and reporting criteria changed substantially, including a period of restricted notification (weeks 39 of 2022 to 18 of 2023) and the transition to sentinel-based surveillance from week 19 of 2023. To facilitate longitudinal interpretation across these phases, we examined weekly incidence using population-based indicators and applied a bridging approach for the post-transition period as described in the Methods.

### Summary of epidemic wave characteristics

**Fig. 3:**
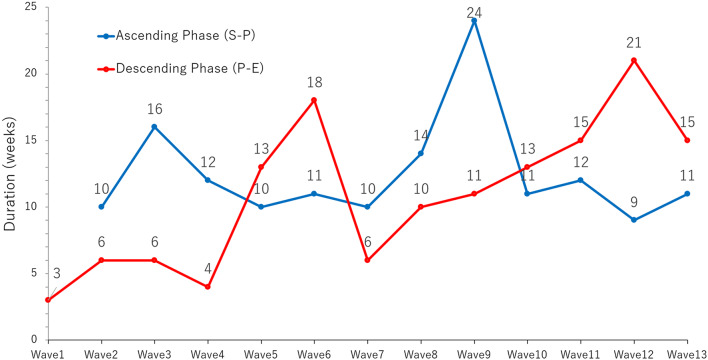
Duration of the ascending and descending phases of 13 COVID-19 epidemic waves in Japan, 2020–2025. For each wave, the number of weeks from wave start (S) to peak (P) and from peak (P) to wave end (E) are shown. Wave start, peak, and end were defined using the operational criteria described in the Methods. This figure provides a visual summary of the indicators reported in Table 2.

Tables 1, 2 and 3 summarize key characteristics of the 13 epidemic waves in Japan, including wave duration, dominant variants and vaccination context (Table 1), phase indicators during the ascending and descending periods of each wave (Table 2), and reported cases, deaths, and observed case fatality ratios where available (Table 3). To improve readability, we added Fig. 3 as a visual summary of the phase indicators reported in Table 2. As shown in Fig. 3, the durations of the ascending (S–P) and descending (P–E) phases varied across epidemic waves. Early epidemic waves (Waves 1–4) tended to be shorter and showed higher observed case fatality ratios, whereas subsequent waves within the period for which death counts were available (Waves 5–9) were generally longer with lower observed case fatality ratios.

**Table 2:** Indicators of the ascending and descending phases of 13 COVID-19 epidemic waves in Japan, 2020–2025.

Wave No.	Duration of the wave	Peak			Changes from start to peak of the wave				Changes from peak to end of the wave			
		Week at peak	Weeks from last peak	Rate at peak (*P*)	Rate at start (S)	(*P*)-(S)	Weeks from S to *P*	Average weekly increase	Rate at end (E)	(*P*)-(E)	Weeks from *P* to E	Average weekly decrease
1	2020, weeks 19–22 (2020 May 6 – June 2)	2020. W19	-	0.4	-	-	-	-	0.2	0.2	4	0.1
2	2020, weeks 23–38 (2020 Jun 3 – Sep 22)	W32	13	6.9	0.2	6.7	9	0.7	2.5	4.4	7	0.6
3	2020, weeks 39–2021, w7 (2020 Sep 23–2021 Feb 23)	2021. W1	22	34.5	2.5	32.0	15	2.1	5.3	29.2	7	4.2
4	2021, weeks 8–23 (2021 Feb 24 – Jun 15)	W19	18	29.1	5.3	23.8	11	2.2	7.9	21.2	5	4.2
5	2021, weeks 24–46 (2021 Jun 16 – Nov 23)	W33	14	120.3	7.9	112.3	9	12.5	0.5	119.8	14	8.6
6	2021, weeks 47–2022, w23 (2021 Nov 24–2022 Jun 14)	2022. W5	24	439.6	0.5	439.1	10	43.9	72.4	367.2	19	19.3
7	2022, weeks 24–39 (2022 Jun 15 – Oct 4)	W33	28	1141.0	72.4	1068.6	9	118.7	234.4	906.6	7	129.5
8	2022, weeks 40–2023, w11 (2022 Oct 5–2023 Mar 21)	2023. W1	20	1543.7	234.4	1309.3	13	100.7	61.8	1481.9	11	134.7
9	2023, weeks 12–46 (2023 Mar 22 – Nov 19)	W35	34	1130.2	61.8	1068.4	23	46.5	107.5	1022.7	11	93.0
10	2023, weeks 47–2024, w18 (2023 Nov 20–2024 May 5)	2024. W5	22	890.3	128.5	761.9	10	76.2	125.1	765.2	13	58.9
11	2024, weeks 19–45 (2024 May 6 – Nov 10)	W30	25	803.8	152.2	651.6	11	59.2	81.0	722.8	15	48.2
12	2024, weeks 46–2025, w23 (2024 Nov 11–2025 Jun 8)	2025. W2	24	390.3	104.7	285.6	8	35.7	49.6	340.7	22	15.5
13	2025, weeks 24–2025, w50 (2025 Jun 9–2025 Dec 14)	2025. W34	32	481.3	49.6	431.7	10	43.2	66.7	414.6	16	25.9

Weekly incidence rates per 100,000 population at the start, peak, and end of each wave are shown, along with absolute changes and average weekly increases and decreases during the ascending and descending phases.

## Discussion

### Principal findings and contribution

Our primary contribution is a comparability-oriented, descriptive framework that bridges Japan’s major surveillance transition in 2023 and supports longitudinal interpretation of epidemic-wave patterns under evolving reporting systems. The incidence indicators after the transition are therefore intended to facilitate continuity of descriptive comparison rather than to represent precise estimates of true infections, and they should be interpreted in light of the assumptions required for bridging. Mortality rates and observed case fatality ratios (CFRs) are reported here as secondary indicators to provide additional context, but they are particularly sensitive to changes in case ascertainment, testing behavior, and reporting scope, as well as to the timing of availability of weekly death data.

Moreover, temporal changes in mortality and observed CFRs likely reflect multiple concurrent influences—variant characteristics, vaccination and booster uptake, accumulation of infection-derived and hybrid immunity, improvements in clinical management, and healthcare system adaptation—rather than a single causal driver. Accordingly, our findings should be interpreted as descriptive patterns observed within specific surveillance contexts, and causal attribution is beyond the scope of this study. These considerations and the resulting interpretive constraints are detailed further in the Limitations section.

### Interpretation under evolving surveillance contexts

This study provides a transparent, comparability-oriented description of 13 COVID-19 epidemic waves in Japan across 2020–2025 while explicitly incorporating major changes in surveillance and reporting. Japan transitioned from comprehensive nationwide notification to restricted reporting and later to sentinel-based surveillance, making direct long-term comparisons challenging without careful attention to case ascertainment. Using weekly population-based indicators, we describe epidemic dynamics across heterogeneous reporting frameworks and document period-specific reporting conditions, including restricted notification and the sentinel transition. The resulting curves represent reported epidemic trends shaped by surveillance design and reporting criteria, not true infection incidence. Because death counts were available only through week 18 of 2023, mortality indicators and observed case fatality ratios could not be assessed after the sentinel transition.

Similar challenges in long-term epidemic interpretation have been documented in other countries where surveillance strategies evolved in response to different phases of the pandemic. The present findings highlight the importance of interpreting epidemic curves in light of the surveillance context in which data were collected.

During the early phase of the pandemic, several East Asian countries, including South Korea and Taiwan, limited early expansion of COVID-19 through a combination of rapid public health responses, including testing and digital tools^13,14^. These approaches relied on information and communication technology–based public health responses that facilitated timely identification and follow-up of cases. In contrast, epidemiological investigations in Japan included conventional administrative and reporting processes operated through local public health centers^15^. This operational structure may have limited the speed of information sharing and case follow-up during the early phase of the pandemic. Differences in institutional frameworks and digital infrastructure may therefore help explain variations in early epidemic trajectories observed among countries in the region.

The epidemiological characteristics of COVID-19 in Japan changed over time, coinciding with the emergence of successive SARS-CoV-2 variants. The sixth through eighth epidemic waves were marked by large increases in reported cases and deaths during the period when the Omicron variant became dominant. Omicron is characterized by high transmissibility, which was reflected in the rapid rise in reported case numbers during these waves. During waves 6–8 (for which death counts were available), a divergence between incidence and mortality trends was observed, with high numbers of reported infections accompanied by relatively low mortality. This pattern underscores the importance of interpreting epidemic indicators alongside information on viral characteristics, population immunity, and surveillance practices, rather than relying solely on case counts.

A marked decline in observed case fatality ratios was noted from the fifth epidemic wave onward, despite substantial increases in reported case numbers. This temporal pattern coincided with expanded vaccination coverage and increasing levels of population immunity, particularly among older adults. High vaccination coverage among elderly populations was maintained during later waves, and lower observed case fatality ratios were recorded even during periods of widespread circulation of the Omicron variant. These observations are consistent with a possible association between vaccination rollout, increasing population immunity, and reduced severity at the population level^16^. However, because this study is descriptive in nature, causal relationships between specific interventions and observed outcomes cannot be established.

Throughout the pandemic, Japan implemented a range of non-pharmaceutical interventions (NPIs), including mask wearing, hand hygiene, avoidance of closed and crowded settings, and temporary restrictions on social activities^17^. The implementation of these measures occurred alongside changes in epidemic patterns, particularly during periods when pharmaceutical interventions were limited^18^. While the relative contribution of individual measures cannot be quantified in this study, the timing of epidemic waves suggests that combinations of behavioral measures, healthcare system adaptations, and vaccination efforts collectively shaped observed trends in incidence and mortality.

### Limitations

This study has several limitations. First, the post-transition bridged national case estimates and incidence indicators rely on assumptions derived from a limited overlap calibration period (weeks 15–18 of 2023) and are intended to facilitate continuity of descriptive, comparability-oriented interpretation across reporting phases rather than to provide precise estimates of the true number of SARS-CoV-2 infections. Changes over time in healthcare-seeking behavior, testing practices, and reporting completeness under sentinel-based reporting could affect the stability of the conversion relationship (including the multiplication factor), and thus post-transition bridged values should be interpreted cautiously.

Second, uncertainty in the bridged indicators is not fully quantified using a formal probabilistic model. Accordingly, we avoid over-precise interpretation of post-transition values and emphasize that the bridged indicators are assumption-dependent comparative measures.

Third, mortality rates and observed case fatality ratios (CFRs) are surveillance-context–dependent indicators. They can be strongly influenced by changes in case ascertainment, testing behavior, and reporting scope; therefore, comparisons across periods with different reporting criteria should be interpreted carefully. In addition, weekly death data were consistently available only through week 18 of 2023, which constrains wave-based summaries of deaths and observed CFRs to waves 1–9.

Finally, our analyses were conducted at the national aggregate level, and uniformly comparable public data structures were not available to support consistent age- or region-stratified analyses across all reporting phases from 2020 to 2025. Future work incorporating stratified analyses and alternative approaches to characterizing uncertainty would further strengthen long-term comparative interpretation under evolving surveillance systems.

## Conclusion

In conclusion, we provide a transparent, comparability-oriented bridging framework that enables longitudinal description and interpretation of COVID-19 epidemic-wave patterns in Japan from 2020 to 2025 across the major surveillance transition implemented in 2023. By explicitly documenting the reporting phases, bridging procedure, and operational wave definitions, this approach supports consistent cross-period interpretation using publicly available national data. Mortality rates and observed CFRs are presented as secondary indicators for context and should be interpreted cautiously in light of surveillance-context dependence, reporting changes, and the limited availability of weekly death data. Overall, our framework facilitates long-term comparative interpretation under evolving surveillance systems while clearly acknowledging the assumptions and limitations inherent to bridging.

## Data Availability

The data used in this study are publicly available from official sources, including the Ministry of Health, Labour and Welfare (Japan) and related national surveillance reports and dashboards. Weekly case and death counts were obtained from publicly released national datasets. In addition to the publicly available source data, the derived weekly estimated incidence time series used for analysis (including the sentinel-to-population conversion outputs) are available from the corresponding author upon reasonable request.
